# Supplementary material for: Photocurable 3D-Printable Systems with Controlled Porosity towards CO2 Air Filtering Applications
Source: Polymers (Basel). 2022 Dec 2;14(23):5265. doi: 10.3390/polym14235265 (PMC9740396; doi:10.3390/polym14235265)
Supplement: Supplementary file 1 [file polymers-14-05265-s001.zip › polymers-2045530-supplementary.pdf]

Photocurable 3D-printable systems with controlled porosity towards CO2 air filtering applications

Annalisa Chiappone [1], Alessandro Pedico [2,3] , Stefania Porcu[4], Candido Fabrizio Pirri[2,3], Andrea Lamberti[2,3] and Ignazio Roppolo[2,3,*]

1 Dipartimento di Scienze Chimiche e Geologiche, Università di Cagliari, Complesso Universitario di Monserrato, S.S. 554 bivio Sestu, 09042 Monserrato, Italy
2 Department of Applied Science and Technology, Politecnico di Torino, C.so Duca Degli Abruzzi 24, 10129, Turin, Italy
3 Center for Sustainable Future Technology @Polito, Italian Institute of Technology, Via Livorno 60, 10144, Turin, Italy
4 Department of Physics, University of Cagliari, S.p. no. 8 Km 0700, 09042 Monserrato, CA, Italy

* Correspondence: ignazio.roppolo@polito.it; Tel.: (0039 -0110907412)

Table S1: 3D printing parameters

Initial – No Dye

	Value	Unit
Light Intensity	25.00	mW/cm^2
Slice Thickness	0.100	mm
Exposure Time	5	s
Burn-In exposure	10	s
Burn-In layers	2	

Light Intensity Decrease – No Dye

	Value	Unit
Light Intensity	10.00	mW/cm^2
Slice Thickness	0.100	mm
Exposure Time	5	s
Burn-In exposure	10	s
Burn-In layers	2	

UVA + RS

	Value	Unit
Light Intensity	40.00	mW/cm^2
Slice Thickness	0.100	mm
Exposure Time	30	s
Burn-In exposure	40	s
Burn-In layers	2	

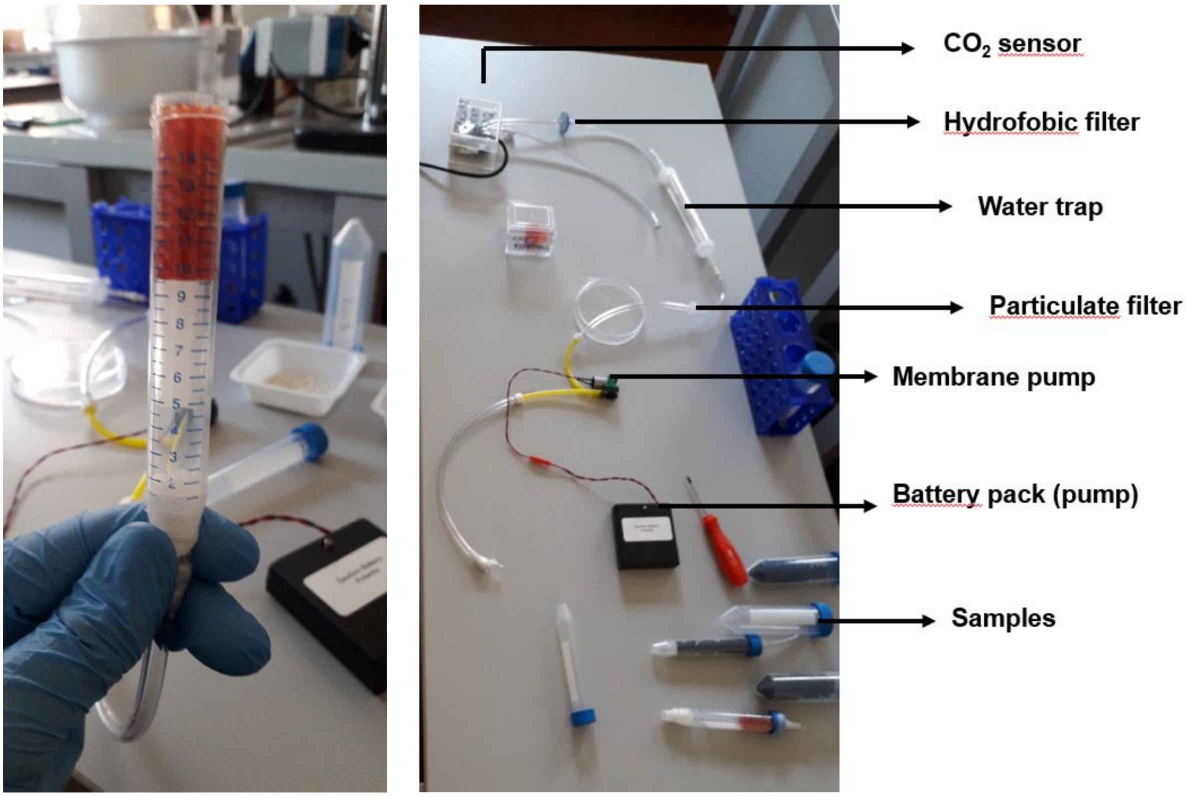

Figure S1. On the left, example of sample inside the cell for CO2 adsorption experiments. On the right, experimental setup for CO2 adsorption.

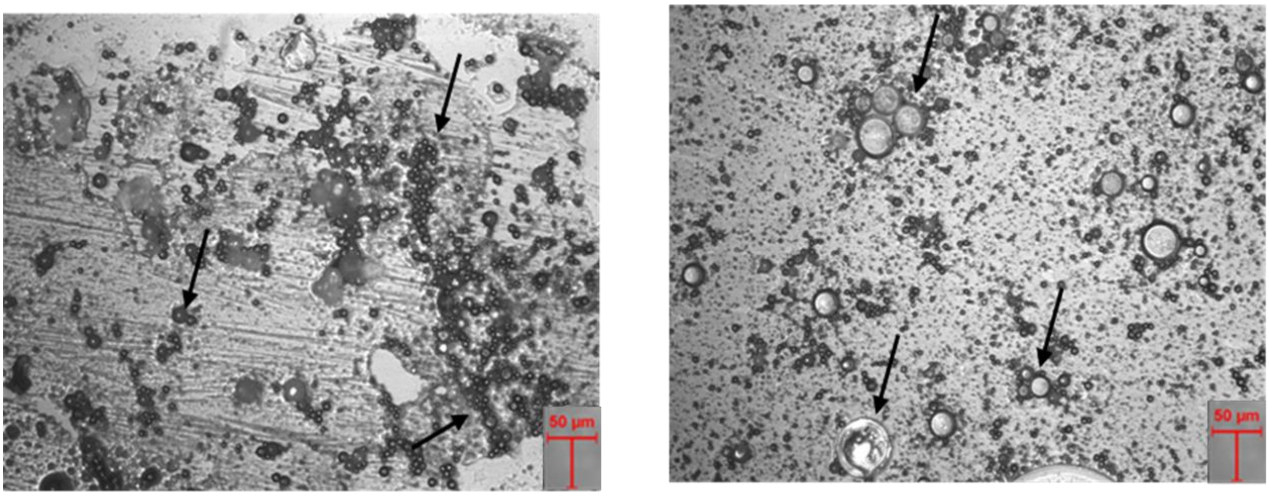

Figure S2 optical of oil in water formulations (#1.5W15.0PL and #1W10.0PL thin films). Arrows indicate photocured polymer spheres dispersed in water.

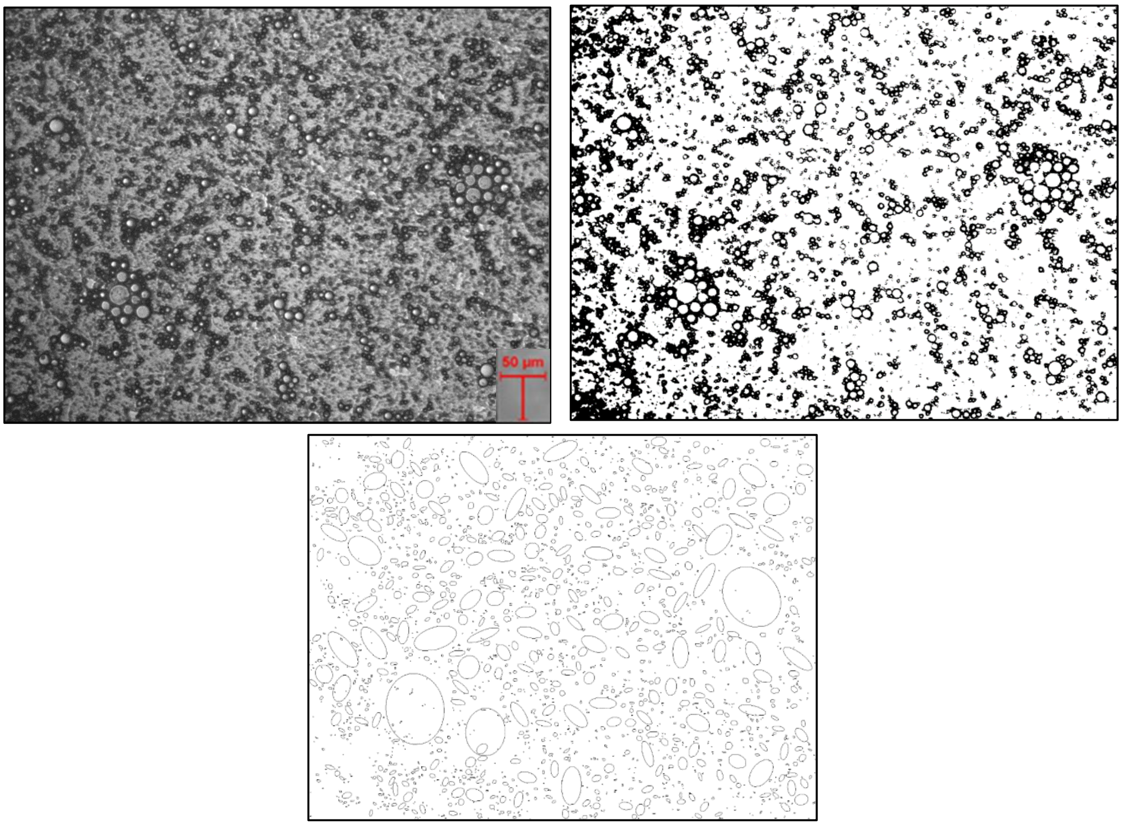

Figure S3: Example of Optical Analysis and relative ImageJ version to calculate the dimension of pores

Table S2: pores average diameter in every formulation

FORMULATION	AVERAGE DIAMETER
#0.5W2.5PL	10.5 µm ± 2 µm
#0.75W3.75PL	8 µm ± 1.5 µm
#0.5W3.75PL	11 µm ± 3 µm
#0.75W5.625PL	12µm ± 3 µm
#0.5W5.0PL	13.5 µm ± 3 µm
#0.75W7.5PL	12.5 µm ± 4 µm

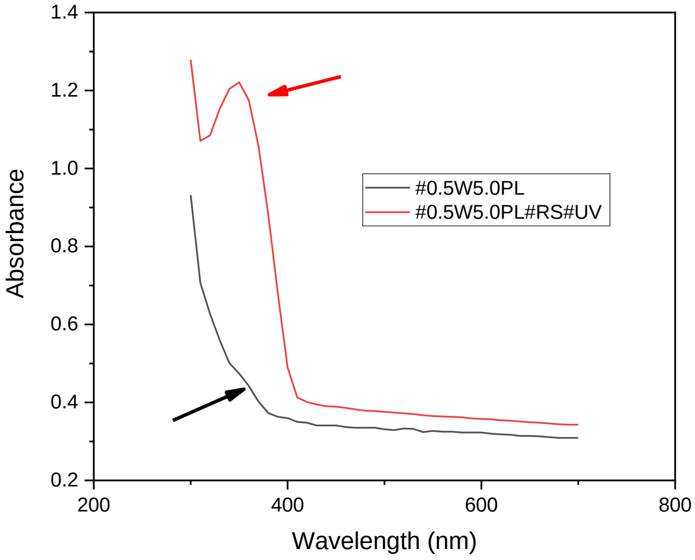

Figure S4: Uv-Vvis spectra showing the absorption of the formulation containing RS and UV compared to initial formulation. Black arrow: absorption peak of the photoinitiator, red arrow, absorption peak of the UV absorber.

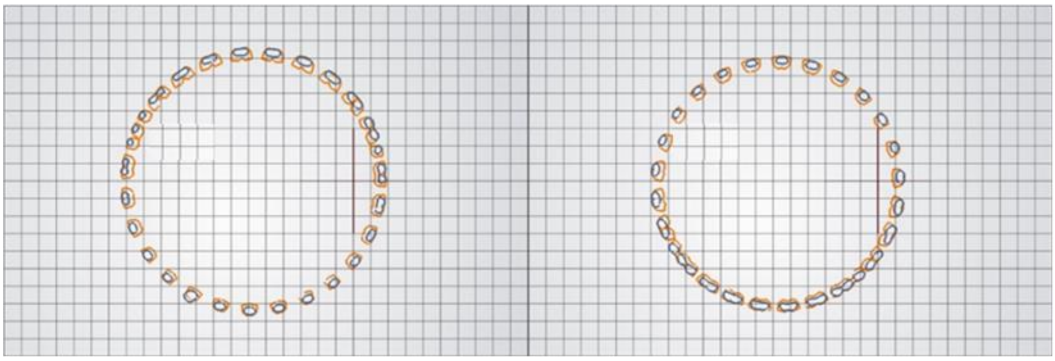

Figure S5: Section of the printed objects in two sections of the object - Comparison between CAD (grey line) and printed object (orange line)

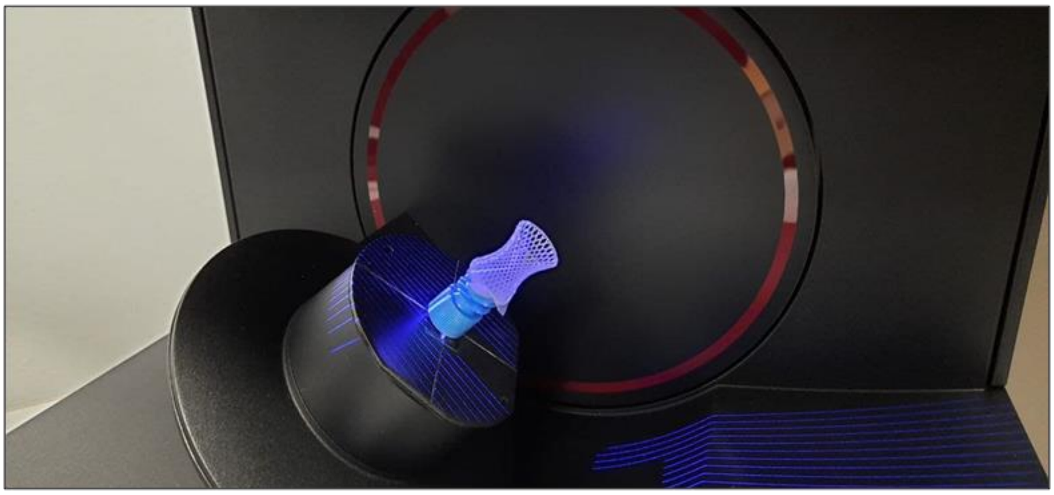

Figure S6: 3D-Scanner experimental setup

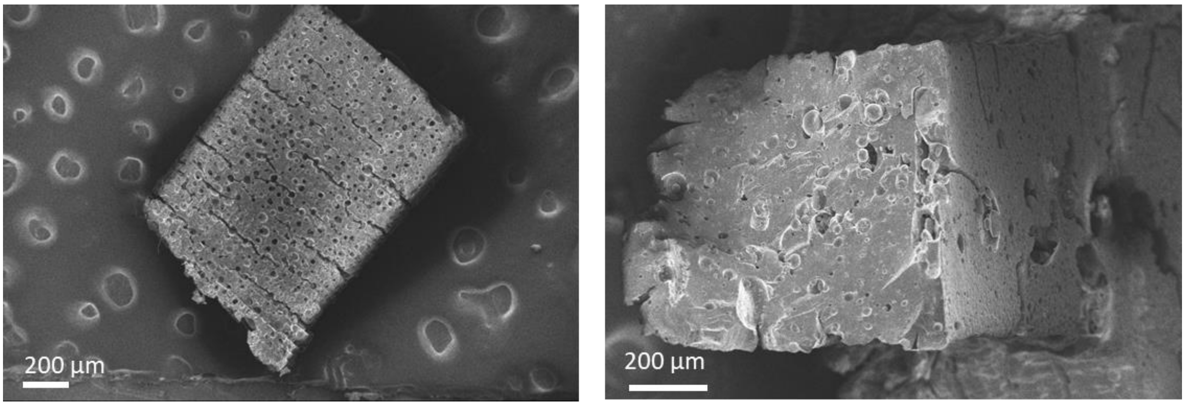

Figure S7 - Samples (#0.5W5.0PL right, #0.75W7.5PL left) structure analysed with SEM (M=400x)

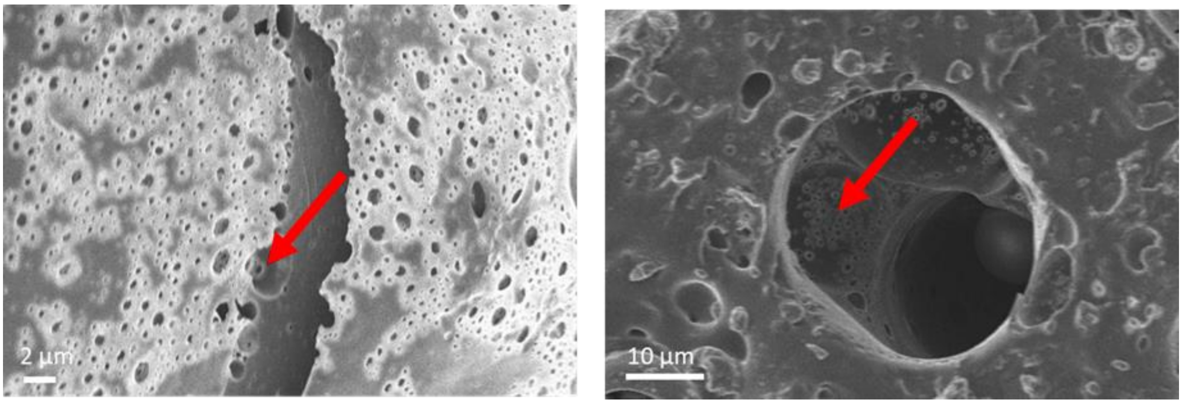

Figure S8 (#0.5W5.0PL#RS#UV left, #0.75W7.5PL#RS#UV right) – Arrows indicated interconnected microporosity in macroporosity.

Table S3 - %DC results

Formulation	% DC
HDDA	77.36
#0.5W5.0PL#RS#UV	74.09
#0.75W7.5PL#RS#UV	73.17

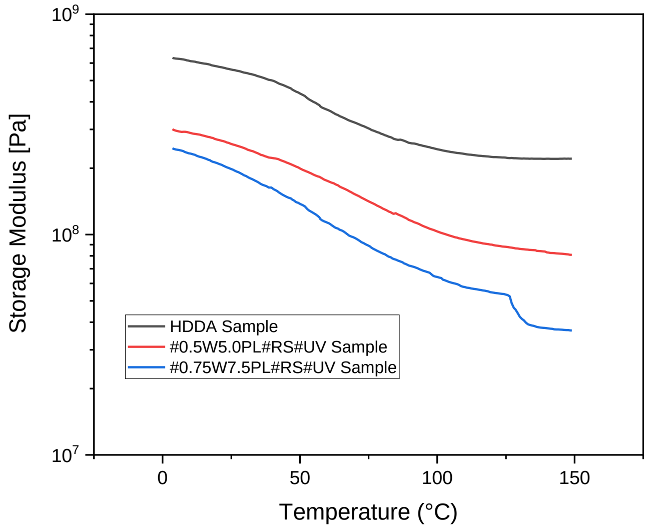

Figure S9 E’ moduli measured in DMTA experiments

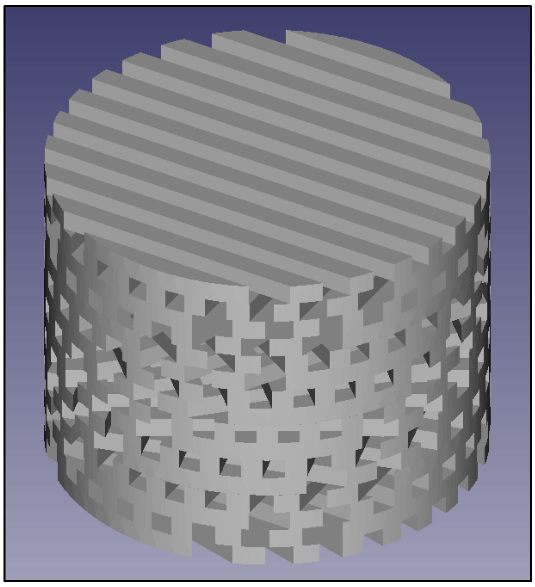

Figure S10 CAD of the filter-like structure

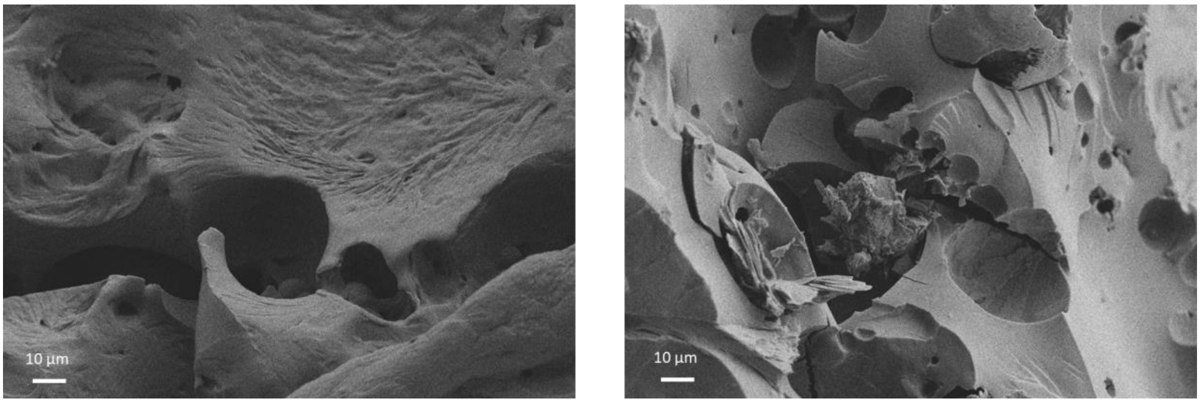

Figure S11 Surface area of 3D printed filter without any treatment
